# Difficulties in Emotion Regulation and Stress in Intensive Care Unit Nurses During COVID-19: Exploring the Mediating Role of Psychological Inflexibility and the Moderating Effect of Work Experience

**DOI:** 10.3390/healthcare13131575

**Published:** 2025-07-01

**Authors:** Cristian Di Gesto, Giulia Rosa Policardo, Sara Bocci Benucci, Eriada Çela, Caterina Grano

**Affiliations:** 1 Department of Psychology, Sapienza University of Rome, Via dei Marsi, 78, 00185 Rome, Italy; 2 Department of Education, Languages, Intercultures, Literatures and Psychology, University of Florence, Via San Salvi, 12, 50135 Florence, Italy; 3 Department of Experimental and Clinical Medicine, University of Florence, Largo Brambilla, 3, 50134 Florence, Italy; 4 Department of Foreign Languages, Faculty of Human Sciences, University of Elbasan “Aleksandër Xhuvani”, Rruga “Ismail Zyma”, 3001 Elbasan, Albania

**Keywords:** emotion regulation, healthcare workers, moderated mediation model, psychological inflexibility, perceived stress

## Abstract

Background/Objectives: The COVID-19 pandemic has placed intensive care unit (ICU) nurses under intense psychological pressure, increasing emotional and psychological stress. Two constructs—difficulties in emotion regulation and psychological inflexibility (i.e., low contact with the present moment and a lack of committed action based on personal values)—have been associated with increased perceived stress levels but remain underexplored in this population. Aims: This study investigated whether psychological inflexibility mediates the relationship between emotion regulation difficulties and perceived stress in ICU nurses. It also examined whether years of ICU work experience moderate the direct relationship between emotion regulation difficulties and perceived stress. Methods: A cross-sectional study was conducted with 210 ICU nurses (65.2% women; 34.8% men; mean age = 40.25 years ± 11.36) from Italian public hospitals. The participants completed the Difficulties in Emotion Regulation Scale, the Acceptance and Action Questionnaire-II, and the Perceived Stress Scale. A moderated mediation model was tested to examine whether psychological inflexibility mediates the relationship between emotion regulation difficulties and perceived stress and whether years of ICU work experience moderate the path between these variables. Results: Higher difficulties in emotion regulation predicted greater psychological inflexibility, which, in turn, predicted higher perceived stress. Psychological inflexibility fully mediated the relationship between emotion regulation difficulties and perceived stress. Additionally, years of ICU work experience significantly moderated the direct link between emotion regulation difficulties and perceived stress. This relationship was strongest for nurses with 1–15 years of ICU experience. The model explained 33% of the variance in perceived stress. Conclusions: This study highlights the importance of the novel construct of psychological inflexibility in the context of healthcare professionals and its role in shaping perceived stress. Addressing psychological inflexibility through targeted interventions may help mitigate stress and promote well-being among ICU nurses.

## 1. Introduction

The unprecedented pressure exerted on healthcare systems by the global SARS-CoV-2 (COVID-19) pandemic has been particularly evident among frontline workers, especially nurses in intensive care units (ICUs). In this high-pressure environment, ICU nurses have been faced with intense emotional demands, long working hours, and an increased risk of infection, all of which contribute to elevated stress levels and a greater psychological burden [1]. Understanding the psychological mechanisms that exacerbate or mitigate this stress is critical for safeguarding nurses’ mental health and ensuring the quality of patient care [2]. Among these mechanisms, emotion regulation and psychological flexibility have emerged as key constructs in explaining individual differences in stress response. However, the interplay between these constructs and the role of occupational factors, such as years of ICU experience, remains underexplored in this population—particularly in countries such as Italy, where ICU nurses faced the first and most intense wave of the pandemic with limited resources and extreme psychological strain [3].

Emotion regulation is a fundamental coping mechanism for stress. When individuals struggle with challenging situations, they may experience overwhelming feelings, react impulsively, and avoid circumstances that trigger negative emotions. Conversely, individuals who are aware of their emotional states and able to regulate their reactions to stressful stimuli typically cope more effectively and report higher levels of overall well-being [4]. While emotion regulation focuses on managing emotional experiences and behavioral responses in specific situations, psychological flexibility encompasses a broader set of adaptive processes that enable individuals to respond effectively to internal experiences over time [5]. In contrast, psychological inflexibility, the opposite facet, is characterized by experiential avoidance (the unwillingness to experience unpleasant thoughts or emotions), cognitive fusion (overidentification with thoughts) and rigid, short-term, emotion-driven behaviors [6].

This notion constitutes a pivotal element within the framework of Acceptance and Commitment Therapy (ACT), a third-wave cognitive-behavioral therapy (CBT) approach [5]. ACT targets six interconnected processes—acceptance (being open to difficult thoughts and emotions), cognitive defusion (seeing thoughts as separate from reality), present-moment awareness (paying attention to the here and now), self-as-context (maintaining a stable sense of self), value clarification (identifying what truly matters), and committed action (taking meaningful steps in line with values)—to enhance psychological flexibility and mitigate inflexibility. Meta-analytic evidence has demonstrated that increased psychological flexibility mediates ACT’s efficacy in treating conditions such as depression, anxiety, and occupational burnout [7,8]. Studying these processes in populations experiencing significant stress, such as healthcare professionals, helps researchers to identify the specific psychological mechanisms and pathways through which interventions based on ACT principles can improve coping strategies and reduce perceived distress in specific work contexts [6,9].

It is important to clearly distinguish psychological flexibility from related constructs. For example, coping styles, as defined by Lazarus and Folkman (1984) [10], describe the specific strategies individuals select (e.g., problem-focused versus emotion-focused coping). In contrast, psychological flexibility concerns how adaptively or rigidly these strategies are employed. From an empirical perspective, psychological inflexibility is a predictor of distress that goes beyond traditional coping styles [11].

Both difficulties in emotional regulation and psychological inflexibility significantly impact healthcare professionals’ well-being, particularly in demanding environments like ICUs. ICUs involve complex patient care, rapid decision-making, and considerable emotional strain, contributing to heightened distress [12,13]. This distress correlates with adverse outcomes such as burnout, reduced job satisfaction [14,15], compromised patient care quality, and negative physical and mental health consequences [16,17].

Additionally, a substantial body of evidence highlights nurses’ mental and emotional states as directly affecting the quality of patient care delivered [18,19]. Enhanced emotional regulation skills can foster psychological flexibility, thereby improving resilience and adaptability in challenging healthcare settings [20].

Several studies have reported that during the COVID-19 pandemic, ICU nurses experienced elevated levels of work-related fatigue, heightened anxiety, distress, and post-traumatic stress disorder (PTSD) [21,22], in addition to higher concerns about infection risk for themselves and their families [23,24]. Worldwide, overall ICU mortality increased from 7.45% in 2019 to 9.4% in 2020 [25].

The COVID-19 pandemic posed additional challenges for Italy, where healthcare professionals confronted a health emergency earlier and more dramatically than in other countries. Scarce resources and excessive work demands further exacerbated healthcare professionals’ distress. Indeed, research has indicated that Italian nurses working in intensive care units during the COVID-19 pandemic faced high levels of burnout, dissatisfaction with their jobs, and psychological distress [26,27]. These factors highlighted the unique and severe challenges that healthcare professionals in Italy encountered throughout this crisis.

Besides the psychological difficulties described above, it is also essential to examine job-related factors, including the number of years of work. Studies investigating the impact of years of work on outcomes such as burnout have yielded mixed findings [28,29,30,31]. Indeed, the relationship between the duration of employment in a specific role and associated outcomes appears to be complex and context-dependent. While some studies suggest a positive correlation between prolonged work experience and perceived stress [30], others report inconclusive or even inverse associations [32]. Despite the pivotal role nurses play in healthcare settings, it is worth highlighting that limited research has explored the role of years of work on the levels of perceived stress in this group.

### The Present Study

Based on the above premises, the present study aims to examine the mediating role of psychological inflexibility in the relationship between difficulties in emotion regulation and stress among Italian nurses, specifically during the unprecedented health challenges of the COVID-19 pandemic. Conducted in the early stages of the pandemic in Italy, one of the first countries to face the crisis with limited access to protective devices and resources, this investigation provides a critical context for understanding how these psychological factors interact.

We hypothesize that greater difficulties in emotional regulation predicted greater perceived stress among Italian nurses, both directly and indirectly, through the mediation of psychological inflexibility. In addition, this research tests the moderating role of years of work in the relationship between difficulties in emotion regulation and perceived stress.

## 2. Materials and Methods

An a priori power analysis was conducted using G * Power [33] version 3.1.9.7 to determine the sample size adequacy. The results indicated that 160 participants would be necessary to achieve a power of 0.95 by assuming a medium effect size (f^2^ = 0.15) and an alpha level of 0.05. Then, a cross-sectional study was conducted with a sample of 210 Italian nurses (65.2% women, 34.8% men; mean age = 40.25 years, standard deviation = 11.36; age range = 25–62 years) working in intensive care units of Italian public hospitals. Initially, approximately 400 ICU nurses were invited to participate in the study. Of these, 318 accessed the online questionnaire, but only 210 provided informed consent and completed all sections of the survey; these participants were included in the final sample.

Participants were recruited using a non-probability convenience sampling method, combined with convenience sampling. Recruitment was conducted through two main channels: (1) the volunteer association Misericordie della Toscana, a regional emergency service organization with strong connections to ICU personnel in public hospitals, and (2) advertisements posted in dedicated social media groups and online forums for nurses working in intensive care settings (e.g., Facebook groups for ICU professionals).

Participants were eligible if they self-declared to be currently working as nurses in the ICUs of public hospitals in Italy. All the participants were informed that their participation was voluntary and anonymous, and confidentiality was guaranteed.

Data were collected between December 2020 and February 2021, during the second wave of the COVID-19 pandemic in Italy.

Participants were invited to take part in the study via an online survey link. On the initial landing page, they were provided with an overview of the study’s objectives, which aimed to explore certain work-related experiences of nurses employed in Italian public intensive care units. Individuals aged 18 and above who provided informed consent were automatically directed to a second page that included questions about demographic characteristics and professional background (e.g., number of years spent working in ICU settings). Following this, the participants completed a series of self-report questionnaires. No financial compensation was offered for participation. The study complied with the ethical standards outlined in the Declaration of Helsinki and received approval from the Institutional Review Board of the University of Sapienza University of Rome (prot. n. 648, 22 April 2020).

### 2.1. Measures

A structured questionnaire was developed for the present study, comprising a set of self-report measures that had been previously validated within the Italian context.

#### 2.1.1. Difficulties in Emotional Regulation

To assess difficulties in emotion regulation, we employed the validated 36-item Italian version [34] of the Difficulties in Emotion Regulation Scale (DERS) [35]. The participants rated each item using a 5-point Likert scale ranging from 1 (almost never) to 5 (almost always). An example item is “I am clear about my feeling.” Elevated scores reflect greater difficulties in regulating emotional responses. In the current sample, internal consistency was excellent (Cronbach’s *α* = 0.93; McDonald’s *ω* = 0.94).

#### 2.1.2. Perceived Stress

Perceived stress was assessed using the 10-item Italian version [36] of the Perceived Stress Scale (PSS) [37]. Items were rated on a 5-point Likert scale from 0 (never) to 4 (very often). A sample item is “In the last month, how often have you been upset because of something that happened unexpectedly?” Higher scores denote higher levels of perceived stress. In our sample, reliability coefficients were acceptable (Cronbach’s *α* = 0.85; McDonald’s *ω* = 0.86).

#### 2.1.3. Psychological Inflexibility

The 10-item Italian version [38] of the Acceptance and Action Questionnaire-II [6] was used to measure psychological inflexibility. Items are presented on a 7-point Likert scale ranging from 1 (it is never true) to 7 (it is always true). A sample item is “Emotions cause problems in my life”. Higher scores indicate higher psychological inflexibility. In the current sample, Cronbach’s alpha and McDonald’s omega were α = 0.85 and ω = 0.85.

#### 2.1.4. Years of Work in Intensive Care Units

The participants were asked to indicate how many years they had worked as nurses in intensive care units in Italian public hospitals. Answers were coded as follows: 1 = less than one year, 2 = from 1 to 5 years, 3 = from 6 to 10 years, 4 = from 11 to 15 years, 5 = from 16 to 20 years, and 6 = more than 20 years. This ordinal variable was recoded as a continuous Likert-type variable, consistent with previous literature and to meet the assumptions of the statistical analyses conducted.

### 2.2. Statistical Analysis

Descriptive statistics and bivariate correlations among the study variables were calculated using IBM SPSS Statistics version 28.0 [39]. The hypothesized moderated mediation model was tested using the PROCESS macro (Model 5), as developed by Hayes [40]. In our model, psychological inflexibility was the mediator between difficulties in emotion regulation (independent variable) and perceived stress (dependent variable). Moreover, it was tested whether the number of years of work in the intensive care unit could moderate the association between difficulties in emotional regulation and perceived stress. To assess the statistical significance of the conditional direct and indirect effects, we employed bias-corrected bootstrap confidence intervals (CIs) based on 5000 bootstrap samples. Effects were deemed significant if the 95% CI did not include zero.

## 3. Results

Descriptive statistics regarding the socio-demographic characteristics of the sample are reported in Table 1.

Regarding the participants’ years of work in ICU, 9.50% of them declared to have worked for less than 1 year, 28.6% from 1 to 5 years, 14.80% from 6 to 10 years, 21% from 11 to 15 years, 20% from 16 to 20 years, and the remaining 6.20% for more than 20 years.

Descriptive statistics and Pearson’s correlation among the study variables are presented in Table 2. Significant negative correlations were found between years of work in intensive care units and difficulties in emotion regulation, psychological inflexibility, and perceived stress, indicating that greater ICU work experience is associated with lower scores on these variables. In contrast, significant positive correlations emerged between difficulties in emotion regulation, psychological inflexibility, and perceived stress, suggesting that higher scores on one of these variables tend to be associated with higher scores on the others.

Building on these initial associations, we proceeded to test the hypothesized moderated mediation model, aimed at evaluating both the direct and indirect pathways through which difficulties in emotion regulation affect perceived stress. Specifically, we examined whether psychological inflexibility mediates this relationship and whether ICU work experience moderates the direct effect of emotion regulation difficulties on perceived stress.

### Moderated Mediation Model

Hayes’s [40] SPSS (version 29) macro PROCESS (Model 5), with a 95% bias-corrected CI based on 5000 bootstrap samples, was used to examine the indirect effects of difficulties in emotional regulation and perceived stress through psychological inflexibility. The number of years of work in the ICU was included as a moderator between difficulties in emotional regulation and perceived stress. The indirect effect was considered statistically significant if the CI did not contain zero. 

**Figure 1:**
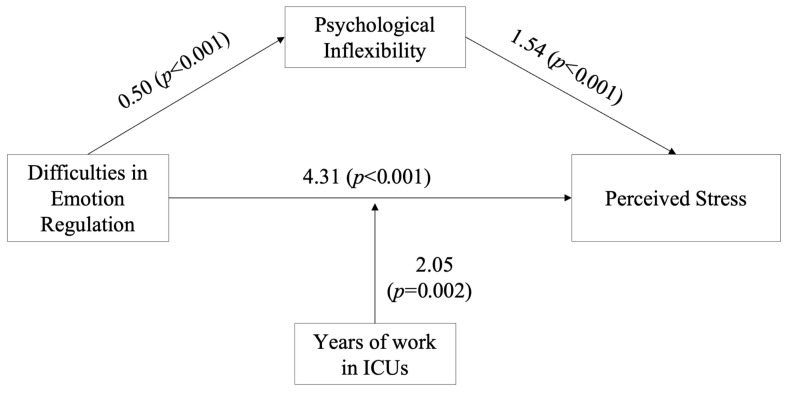
Moderated mediation model. Notes. Coefficients are β unstandardized.

As shown in Figure 1, the results indicate that when nurses report more difficulties in regulating their emotions, they also tend to show higher psychological inflexibility (i.e., difficulty staying in the present and acting in line with personal values), which in turn is associated with higher levels of stress.

Table 3 reports the results of the interaction between difficulties in emotion regulation and years of work in ICU on perceived stress. The effect of emotion regulation difficulties on perceived stress was found to vary depending on the participants’ ICU work experience.

Specifically, among nurses with less than one year to five years of ICU experience, the effect was strong and statistically significant (Effect = 2.28, SE = 0.53, 95% CI [1.23, 3.32]), meaning that greater difficulties in emotion regulation were clearly associated with higher levels of perceived stress in this group. A weaker but still significant effect was found for nurses with six to fifteen years of ICU experience (Effect = 1.26, SE = 0.41, 95% CI [0.45, 2.06]).

In contrast, for nurses with more than 16 years of ICU experience, the relationship between emotion regulation difficulties and perceived stress was not significant (Effect = −0.78, SE = 0.59, 95% CI [−1.95, 0.39]). This suggests that among more experienced nurses, difficulties in emotion regulation were not linked to perceived stress in a statistically meaningful way.

The interaction effect between emotion regulation difficulties and ICU experience was significant overall, as shown in the test of the highest-order unconditional interaction (ΔR^2^ = 0.05, F = 14.94, *p* = 0.0001), indicating that the strength of this relationship truly differs across levels of experience.

These results suggest that ICU work experience may act as a protective factor: the more years nurses have worked in intensive care, the less their emotional difficulties translate into stress. This may reflect the development of emotional coping strategies and professional resilience over time.

Additionally, psychological inflexibility fully mediates the relationship between difficulties in emotion regulation and perceived stress (b = 0.80, SE = 0.20, 95% CI [0.40,1.20]). This means that the direct effect of emotion regulation difficulties on perceived stress becomes non-significant when psychological inflexibility is included in the model. The tested model explains 33% of the variance in perceived stress, indicating a substantial proportion of explained variability in the outcome variable.

## 4. Discussion

The current study aimed to explore the relationship between difficulties in emotion regulation and perceived stress among Italian nurses working in ICUs during the COVID-19 pandemic. Additionally, the study sought to evaluate whether psychological inflexibility acted as a mediator in this relationship. Furthermore, it examined the moderating role of years of experience in the connection between emotional regulation difficulties and perceived stress. The present study’s findings contribute to the relatively underexplored area of psychological inflexibility concerning distress and well-being [41,42], extending the study of this construct within the context of nurses working in ICUs during the COVID-19 pandemic in Italy. Our findings indicated that greater emotional regulation difficulties are associated with perceived stress, both directly and indirectly, through psychological inflexibility.

### 4.1. Summary of Findings

The direct relationship between difficulties in emotional regulation and perceived stress is consistent with previous research. Nurses who experience higher emotional dysregulation may be more prone to feeling overwhelmed, particularly when exposed to critical-care demands during a crisis like the COVID-19 pandemic. Studies from Italy, Brazil, and China likewise show that individuals with poor emotion regulation skills report higher stress levels and emotional burden [43,44,45]. Our moderation analysis further revealed that this relationship was significant primarily for nurses with 15 or fewer years of ICU experience. This suggests that younger or less experienced nurses may possess a more limited repertoire of coping strategies, making it more difficult for them to manage emotionally demanding clinical scenarios. This interpretation is supported by evidence from various cultural and healthcare contexts. For instance, a national study in Iran found that ICU nurses with fewer years of experience reported significantly higher stress levels than their senior colleagues [17]. Similarly, research from the southeastern United States highlighted the profound psychological toll and trauma experienced by early-career nurses caring for COVID-19 patients in intensive care [21]. Mehta et al. [22] showed that ICU staff in Canada with less professional tenure perceived greater emotional strain and reduced resilience in response to pandemic-related stressors. These findings, consistent with our results, underscore the specific vulnerability of less experienced nurses when facing high-intensity clinical environments.

Cross-national comparisons point to organizational features that may amplify—or buffer—these individual risks. For example, in Italy and Spain, ICUs often operate with nurse-to-patient ratios of approximately 1:3 to 1:4 [46,47], whereas guidelines in the UK, Australia, and Nordic countries recommend 1:1 to 1:2 coverage for ventilated patients [48]. Lower ratios (≤1.5:1) have been associated with reduced burnout and lower ICU mortality in large international cohorts [49]. Similarly, working 12-hour shifts is associated with greater fatigue and psychological distress than working 8-hour shifts [50,51]. These systemic differences help explain international variability in stress outcomes and add a critical context to our findings.

Drawing on Lazarus and Folkman’s (1984) [10] Stress and Coping Framework, it is plausible that more experienced nurses develop adaptive strategies over time that buffer the impact of emotional dysregulation. This phenomenon was likely exacerbated during the COVID-19 pandemic, when many healthcare systems rapidly deployed less experienced nurses to address staffing shortages, thereby exposing them to unprecedented psychological strain [19,23].

Considering the indirect pathway, psychological inflexibility significantly mediated the link between emotion regulation difficulties and perceived stress. This is consistent with the ACT framework [5]. Internationally, Chong et al. [52] found that inflexibility mediated burnout and mental health symptoms among Chinese nurses; Smith et al. [53] reported that inflexibility and intolerance of uncertainty amplified mental health decline among U.S. staff; and Jiménez-Fernández et al. [54] documented a strong relationship between inflexibility and burnout in Spanish frontline nurses. Despite varied contexts, all studies converge on inflexibility as a robust predictor of distress, supporting our mediation model. Furthermore, Hayes et al.’s psychological flexibility model posits that experiential avoidance and cognitive fusion maintain and amplify emotional distress under chronic stress—especially in emotionally intense and ethically complex ICU contexts [5,6].

Overall, our findings highlight psychological inflexibility as a key mechanism linking emotion regulation challenges to elevated stress. Interventions targeting flexibility (e.g., ACT, brief mindfulness) should be implemented alongside system-level policies—such as optimal nurse-to-patient ratios, evidence-based shift scheduling, and accessible mental health support—adapted to local organizational and cultural contexts. Such multilevel strategies may be crucial to safeguard ICU nurses’ well-being in high-pressure healthcare environments.

### 4.2. Practical Implications

Given the observed mediating role of psychological inflexibility, interventions that target this mechanism could play a pivotal role in alleviating stress among ICU nurses. While only a single study, in China, has directly evaluated group-based ACT in ICU nurses, broader evidence from general and physician populations reinforces its applicability. Empirical evidence supports the efficacy of ACT across healthcare settings. For example, Han et al. [55] demonstrated improved psychological well-being in nurses through a four-session group-based ACT program, while Smith et al. [53] found that brief ACT training significantly reduced emotional exhaustion among physicians. Furthermore, Fledderus et al. [7] reported reductions in psychological inflexibility and distress through self-guided ACT protocols, and Chong et al. [52,56] observed similar benefits in community samples.

In high-pressure ICU environments, it is crucial to have scalable and adaptable strategies. Brief mindfulness interventions, such as five-minute ACT-consistent practices during shift changes, have shown promise in improving emotional regulation and reducing stress in healthcare providers [57]. A structured peer support program [58] informed by ACT principles may facilitate collective emotional processing, value clarification, and improved team functioning. Furthermore, leadership development initiatives that equip ICU managers with ACT-informed communication and support skills could foster greater psychological safety and engagement among staff. A recent study by Blanco-Donoso et al. [59] showed that participants who completed an ACT program experienced significant reductions in depressive symptoms and stress, with these benefits persisting at follow-up.

These interventions can be integrated into existing organizational structures, such as weekly staff meetings or digital learning platforms, requiring minimal additional resources while maximizing reach and scalability. Furthermore, the effectiveness of such interventions may vary according to the level of experience of the nurse. Our findings suggest that less experienced nurses are particularly vulnerable to the effects of emotional dysregulation on stress, indicating that they could benefit most from targeted interventions designed to enhance psychological flexibility early in their careers. Future research should therefore explore whether tailoring ACT-based modules by experience level can optimize intervention outcomes and support sustainable workforce resilience in critical care settings.

### 4.3. Limitations

Several methodological limitations must be acknowledged. The cross-sectional design of this study prevents causal interpretations, so future longitudinal studies are recommended to clarify directionality. Furthermore, potential covariates such as personality traits (e.g., neuroticism and conscientiousness) and pre-existing mental health conditions (e.g., anxiety and depression) were not controlled for, even though they might theoretically influence stress perception and emotional regulation capabilities. Individuals may be more likely to experience heightened emotional responses or maladaptive coping mechanisms due to their personality traits, potentially intensifying their perceived stress levels. Additionally, a pre-existing mental health history can independently contribute to heightened stress vulnerability, potentially obscuring the observed relationships [60].

In addition, the absence of a control or comparison group limits the study’s internal validity and replicability. Including a control group (or data from a small pilot study conducted after the pandemic) would have enabled a more accurate assessment of changes in psychological variables over time. However, given the time-sensitive and rapidly evolving nature of the context of the pandemic, it was not feasible to implement such conditions. Future research should incorporate control groups or longitudinal pilot designs to strengthen causal inference and replicability.

Another consideration is the potential for common method bias and endogeneity inherent in self-report measures and correlational designs. The use of psychometrically distinct scales, anonymity, and the assurance of no right/wrong answers were employed to mitigate common method bias. However, reverse causality or omitted variable bias cannot be ruled out due to the cross-sectional nature. Future experimental or longitudinal designs are necessary to robustly address these endogeneity concerns.

Furthermore, we did not perform subgroup analyses on socio-demographic variables such as gender or education level. While our primary aim focused on the mediating role of psychological inflexibility and the moderating effect of ICU experience, gender imbalance (predominantly female sample) is typical in nursing research and may influence generalizability. Future studies should examine whether socio-demographic factors moderate the observed relationship.

Finally, the specific sample of Italian ICU nurses during the pandemic may have compromised the generalizability of the results. Cross-cultural comparisons could help us understand the role of psychological flexibility more fully around the world and across different healthcare systems. Going forward, it would be worthwhile to try to replicate these findings by including nurses in ICUs during periods when there are no emergencies, such as those not related to public health crises like the pandemic caused by the SARS-CoV-2 virus. Nevertheless, the study’s unique and unprecedented ecological situation of chronic stress is also its strength.

## 5. Conclusions

This study examined the relationships between emotion regulation difficulties, psychological inflexibility, and perceived stress in ICU nurses during the COVID-19 pandemic, offering evidence for the role of psychological inflexibility as a core mechanism linking emotional dysregulation to stress. These findings extend the theoretical relevance of the Psychological Flexibility Model [5], highlighting its utility in understanding stress responses in occupational settings and under chronic crisis conditions, such as public health emergencies.

From a managerial perspective, the results suggest that healthcare organizations should adopt structured and preventive approaches to support the psychological well-being of their staff. In particular, the integration of ACT-informed programs into institutional policies, the inclusion of emotion regulation and psychological flexibility training in continuing professional development, and the implementation of regular assessments to monitor staff well-being may prove valuable. Attention should especially be directed to younger or less experienced nurses, who appear to be more vulnerable to the effects of emotional dysregulation and rigidity in stressful contexts. These strategies could promote more adaptive coping, reduce burnout risk, and improve the sustainability of the healthcare workforce over time.

Given the correlational nature of this study, future research should adopt longitudinal and experimental designs to clarify the causal pathways linking emotion regulation, psychological inflexibility, and perceived stress. Further investigations might also explore the role of contextual factors such as leadership style, team dynamics, and organizational support in shaping these psychological processes. Expanding the scope to non-pandemic conditions and different healthcare systems would also be important to assess the generalizability of the findings and to better understand how structural and cultural variables interact with individual psychological traits. Overall, this study points to psychological inflexibility as a key theoretical construct and practical intervention target in the promotion of mental health among healthcare professionals operating in highly demanding environments.

## Figures and Tables

**Table 1:** Socio-demographic characteristics and information about how long they have worked as a nurse.

Socio-Demographic Characteristics		%
Education level		
	Bachelor’s degree	37.6%
	Master’s degree	41.8%
	Post lauream specialization courses	12.3%
	Ph.D.	8.3%
Marital status		
	Unmarried	49.8%
	Married/cohabiting	42.5%
	Separated/divorced	7.7%
Place of residence		
	Northern Italy	5.5%%
	Central Italy	80.8%
	Southern Italy or Islands	13.7%
Occupation		
	Full-time	54.6%
	Part-time	45.4%

**Table 2:** Descriptive statistics and correlations among the study variables (N = 210).

	1.	2.	3.	4.	*M* (*SD*)
1. Years of work in ICU					3.31 (1.46)
					
2. Difficulties in emotion regulation	−0.23				2.55 (0.68)
	*p* = <0.001				
3. Psychological inflexibility	−0.16	0.29			3.22 (1.70)
	*p* = 0.018	*p* = <0.001			
4. Perceived stress	−0.23	0.30	0.50		3.38 (4.48)
	*p* = <0.001	*p* = <0.001	*p* = <0.001		

Notes. Years of work in intensive care units was measured as an ordinal variable and recoded as a continuous Likert-type variable for statistical purposes. Coding: 1 = less than a year; 2 = 1–5 years; 3 = 6–10 years; 4 = 11–15 years; 5 = 16–20 years; 6 = more than 20 years.

**Table 3:** Conditional effects.

Difficulties in Emotional Regulation * Years of Work in ICU (X * W)							
**Years of Work in ICU**	**Effect**	**se**	**LLCI**	**ULCI**	*R^2^* change X * W	***F***	***p***
Less than a year to five years	2.27	0.53	1.23	3.31			
Six to fifteen years	1.26	0.41	0.45	2.05			
Over sixteen years	−0.78	0.59	−1.95	0.38			
Test of highest-order unconditional interaction					0.05	14.94	0.0001
**Conditional effect of difficulties in emotional regulation on perceived stress**							
**Years of Work in ICU**	**Effect**	**se**	**LLCI**	**ULCI**			
Less than a year to five years	2.28	0.53	1.23	3.32			
Six to fifteen years	1.26	0.41	0.45	2.06			
Over sixteen years	−0.78	0.59	−1.95	0.39			

Notes. *SE* = standard error; *LLCI* = lower-limit confidence interval; *ULCI* = upper-limit confidence interval; Δ*R^2^* = change in explained variance due to the interaction term.

## Data Availability

The fully anonymized datasets analyzed during this study are available from the corresponding author upon reasonable request, with no additional privacy or ethical restrictions.
